# A guide to selecting high-performing antibodies for VCP (UniProt ID: P55072) for use in western blot, immunoprecipitation, and immunofluorescence

**DOI:** 10.12688/f1000research.169502.1

**Published:** 2025-09-09

**Authors:** Vera Ruíz Moleón, Maryam Fotouhi, Joel Ryan, Donovan Worrall, Riham Ayoubi, Vincent Francis, Peter S McPherson, Carl Laflamme

**Affiliations:** 1 Neuroscience and Neurosurgery, McGill University, Montreal, Québec, Canada; 2 McGill University, Montreal, Québec, Canada

**Keywords:** P55072, VCP, Valosin-containing protein, Transitional endoplasmic reticulum ATPase, antibody characterization, antibody validation, western blot, immunoprecipitation, immunofluorescence

## Abstract

Valosin-containing protein (VCP) is a highly conserved and essential ATPase involved in many cellular processes like neuronal function, protein degradation, organelle maintenance, and stress response regulation. Understanding the specific mechanisms by which VCP plays in health and disease can provide novel insides in therapeutic targets, a process that would be facilitated by the availability of high-quality antibodies. Here we have characterized sixteen VCP commercial antibodies for western blot, immunoprecipitation, and immunofluorescence using a standardized experimental protocol based on comparing read-outs in knockout cell lines and isogenic parental controls. These studies are part of a larger, collaborative initiative seeking to address antibody reproducibility issues by characterizing commercially available antibodies for human proteins and publishing the results openly as a resource for the scientific community. While the use of antibodies and protocols vary between laboratories, we encourage readers to use this report as a guide to select the most appropriate antibodies for their specific needs.

## Introduction

VCP is a member of the AAA+ (ATPases Associated with diverse cellular Activities) family and a highly conserved ATPase that plays a pivotal role in multiple cellular processes that are essential for neuronal function, including protein degradation, organelle maintenance, and stress response regulation. ^1– 3^ This protein is a key component of the ubiquitin-proteasome system and autophagy pathways, facilitating the extraction and degradation of misfolded or aggregated proteins from various subcellular compartments. ^4^ In the central nervous system, the maintenance of proteostasis is critical for neuronal survival, synaptic integrity, and plasticity. Mutations in the *VCP* gene have been implicated in a spectrum of neurodegenerative diseases, including the inclusion body myopathy with Paget’s disease and frontotemporal dementia (IBMPFD), amyotrophic lateral sclerosis (ALS), and Parkinson’s disease. ^5– 7^ These pathogenic variants disrupt the VCP’s function, leading to impaired protein clearance, mitochondrial dysfunction, and aberrant stress granule dynamics, ultimately contributing to neuronal degeneration. Recent studies also highlight the involvement of VCP in modulating neuroinflammation and axonal transport, underscoring its multifaceted roles in neural homeostasis. ^8^ Understanding the mechanistic underpinnings of VCP dysfunction offers promising avenues for targeted therapeutic interventions in neurodegenerative disorders.

This research is part of a broader collaborative initiative in which academics, funders and commercial antibody manufacturers are working together to address antibody reproducibility issues by characterizing commercial antibodies for human proteins using standardized protocols and openly sharing the data. ^9^ Here we characterized sixteen commercial VCP antibodies, selected and donated by participant antibody manufacturers, for use in western blot, immunoprecipitation, and immunofluorescence (also referred to as immunocytochemistry), enabling biochemical and cellular assessment of VCP properties and function.

The authors do not engage in result analysis or offer explicit antibody recommendations. Our primary aim is to deliver top-tier data to the scientific community, grounded in Open Science principles. This empowers experts to interpret the characterization data independently, enabling them to make informed choices regarding the most suitable antibodies for their specific experimental needs. Guidelines on how to interpret antibody characterization data found in this study are featured on the YCharOS gateway ^10^ and in Table 4 of this data note. ^9^

## Results and discussion

Our standard protocol involves comparing readouts from wild type (WT) and KO cell lines. ^11^ In the absence of commercially available KO cell lines, siRNA technology can be employed to knockdown (KD) the target gene. ^12, 13^ As *VCP* is an essential gene in many cancer cells, a knockdown (KD) approach was used to deplete the corresponding mRNA using siRNA. ^13^ To determine which cell line demonstrates high expression of VCP protein and thus be appropriate for KD, the first step is to identify a cell line that expresses sufficient levels of a given protein to generate a measurable signal using antibodies. To this end, we examined the DepMap (Cancer Dependency Map Portal, RRID:SCR_017655) transcriptomics database to identify cell lines that express the target at levels greater than 2.5 log _2_ (transcripts per million “TPM” + 1), which we have found to be a suitable cut-off. ^14^ We selected the U-2 OS cell line for this study. A non-targeting control siRNA pool was used to treat U-2 OS control (ctrl) cells, while *VCP* was KD using a pool of siRNA targeting this gene.

**Figure 1.**
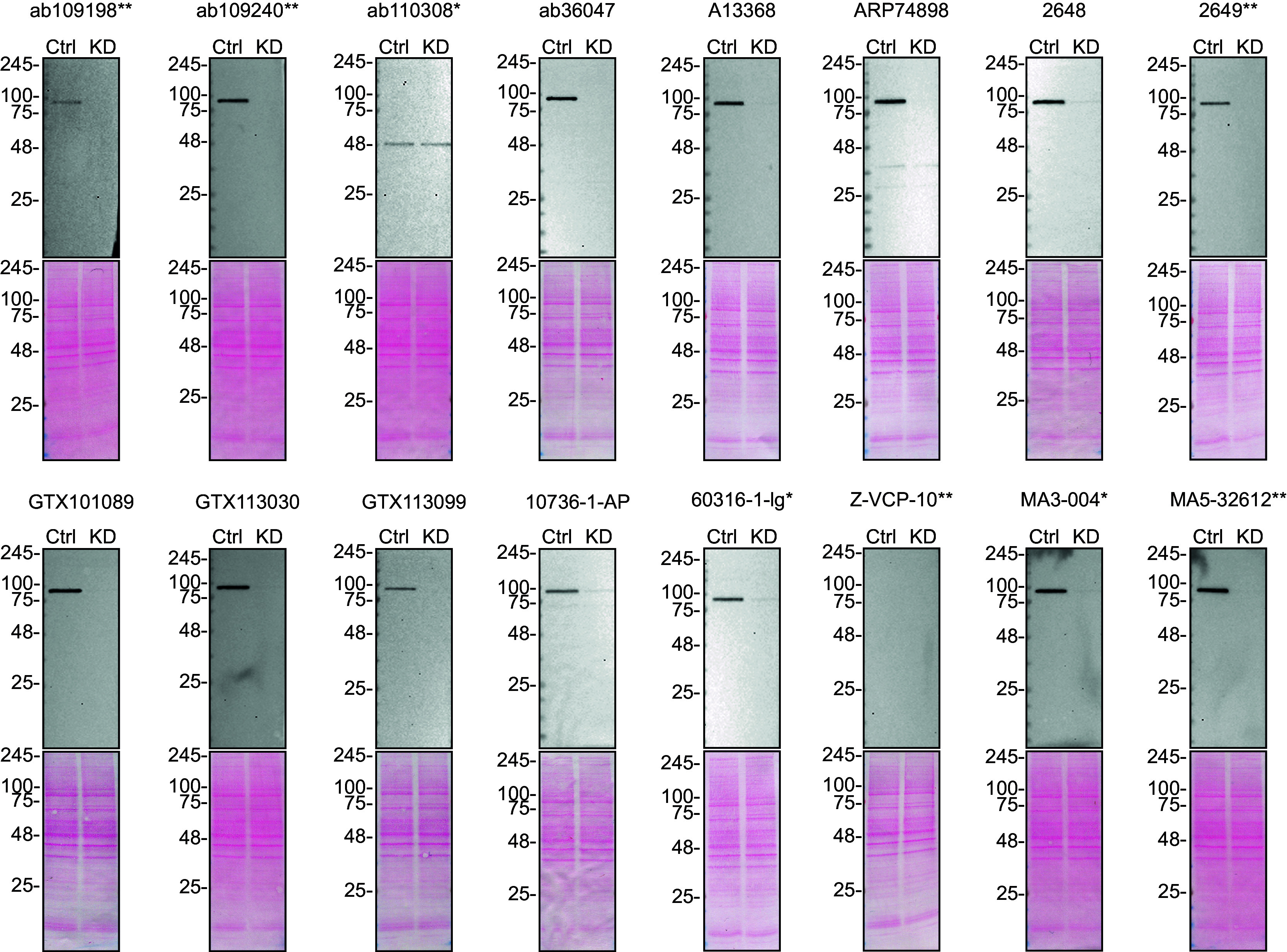
VCP antibody screening by western blot. Lysates of U-2 OS ctrl and *VCP* KD were prepared, and 10 μg of protein were processed for western blot with the indicated VCP antibodies. The Ponceau stained transfers of each blot are presented to show equal loading of ctrl and KD lysates and protein transfer efficiency from the acrylamide gels to the nitrocellulose membrane. Antibody dilutions were chosen according to the recommendations of the antibody supplier. Antibody dilution used: ab109198** at 1/10 000; ab109240** at 1/50 000; ab110308* at 1/10 000; ab36047 at 1/10 000; A13368 at 1/10 000; ARP74898 at 1/10 000; 2648 at 1/10 000; 2649** at 1/10 000; GTX101089 at 1/50 000; GTX113030 at 1/50 000; GTX113099 at 1/50 000; 10736-1-AP at 1/15 000; 60316-1-lg* at 1/10 000; Z-VCP-10** at 1/1000; MA3-004* at 1/30 000; MA5-32612** at 1/30 000. Predicted band size: 89 kDa. ** = recombinant antibody, * = monoclonal antibody.

To screen all sixteen antibodies by western blot, U-2 OS ctrl and *VCP* KD protein lysates were ran on SDS-PAGE, transferred onto nitrocellulose membranes, and then probed with the sixteen antibodies in parallel ( Figure 1).

**Figure 2.**
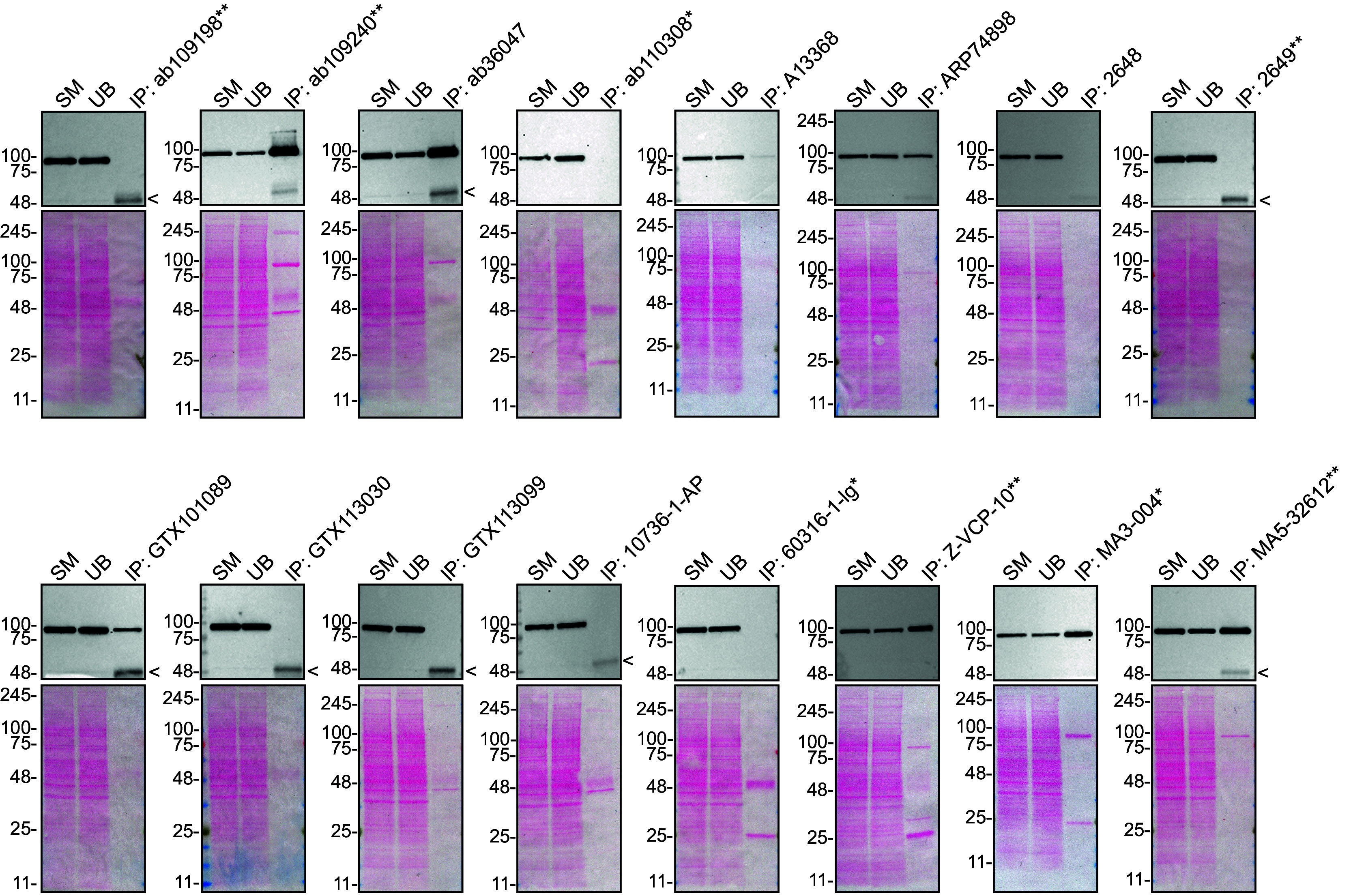
VCP antibody screening by immunoprecipitation. U-2 OS lysates were prepared, and immunoprecipitation was performed using 0.5 mg of protein and 2.0 μg of the indicated VCP antibodies pre-coupled to Dynabeads protein A, protein G or Flag-M2 magnetic beads. Samples were washed and processed for western blot with the indicated VCP antibody. For western blot, MA3-004* was used at 1/1000. The Ponceau stained transfers of each blot are shown. SM = 4% starting material, UB = 4% unbound fraction, IP = immunoprecipitate, <=points toward the antibody heavy chain, ** = recombinant antibody, * = monoclonal antibody.

We then assessed the capability of all sixteen antibodies to capture VCP from U-2 OS protein extracts using immunoprecipitation techniques, followed by western blot analysis. For the immunoblot step, a specific VCP antibody identified previously (refer to Figure 1) was selected. Equal amounts of the starting material (SM) and the unbound fractions (UB), as well as the whole immunoprecipitate (IP) eluates were separated by SDS-PAGE ( Figure 2).

**Figure 3.**
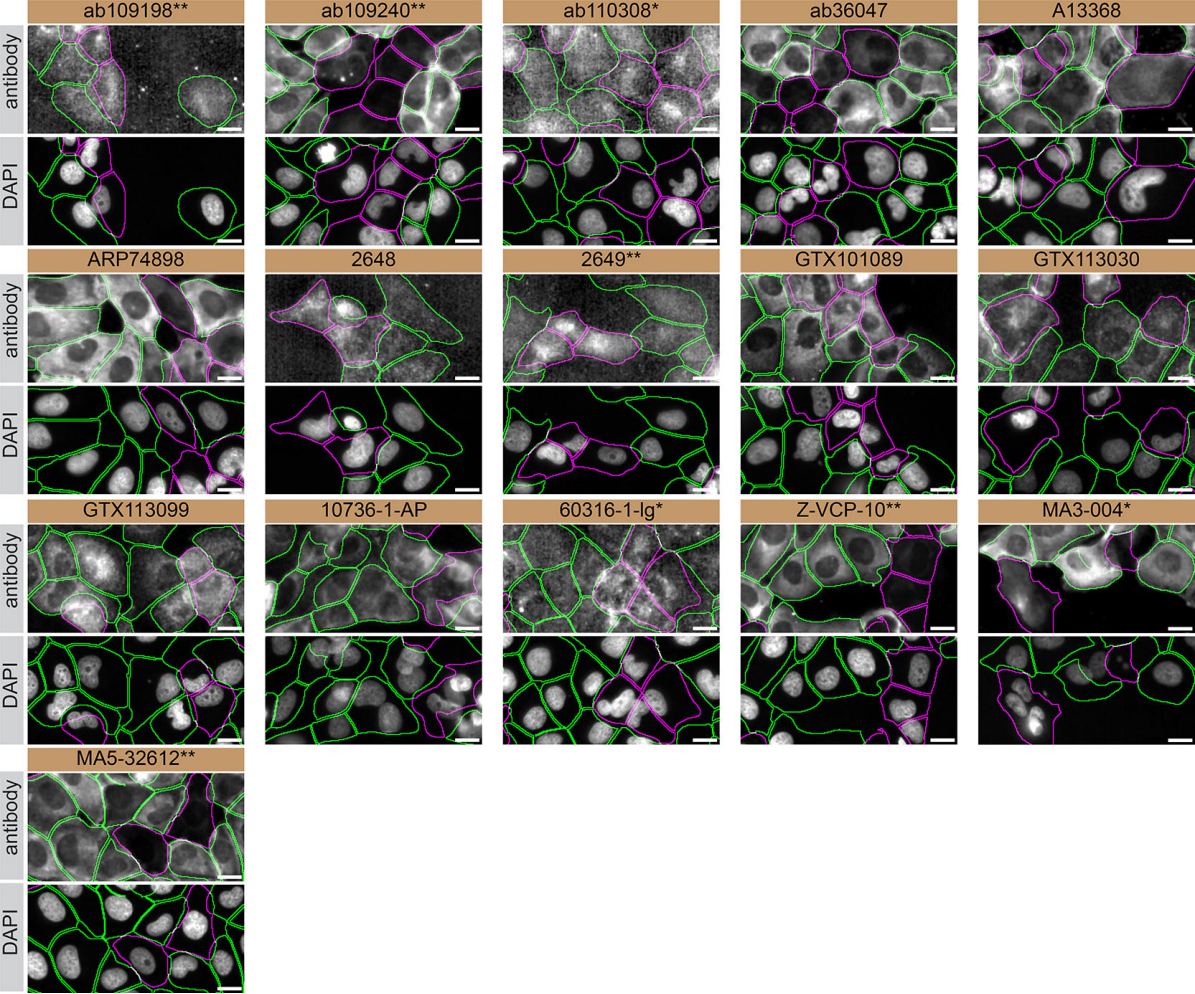
VCP antibody screening by immunofluorescence. U-2 OS ctrl and *VCP* KD cells were labelled with a green or a far-red fluorescent dye, respectively. Ctrl and KD cells were mixed and plated to a 1:1 ratio on coverslips. Cells were stained with the indicated VCP antibodies and with the corresponding Alexa-fluor 555 coupled secondary antibody including DAPI. Acquisition of the blue (nucleus-DAPI), green (ctrl), red (antibody staining) and far-red (KD) channels was performed. Representative images of the blue and red (grayscale) channels are shown. Ctrl and KD cells are outlined with green and magenta dashed line, respectively. When an antibody was recommended for immunofluorescence by the supplier, we tested it at the recommended dilution. The rest of the antibodies were tested at 1 and 2 μg/ml, and the final concentration was selected based on the detection range of the microscope used and a quantitative analysis not shown here. Antibody dilution used: ab109198** at 1/800; ab109240** at 1/500; ab110308* at 1/1000; ab36047 at 1/100; A13368 at 1/100; ARP74898 at 1/100; 2648 at 1/100; 2649** at 1/100; GTX101089 at 1/500; GTX113030 at 1/500; GTX113099 at 1/1000; 10736-1-AP at 1/1000; 60316-1-lg* at 1/50; Z-VCP-10** at 1/100; MA3-004* at 1/1000; MA5-32612** at 1/1000. Bars = 10 μm. ** = recombinant antibody, * = monoclonal antibody.

For immunofluorescence, the sixteen antibodies were screened using a mosaic strategy. First, U-2 OS ctrl and *VCP* KD cells were labelled with different fluorescent dyes in order to distinguish the two cell lines, and the VCP antibodies were evaluated. Both ctrl and KD lines imaged in the same field of view to reduce staining, imaging and image analysis bias ( Figure 3). Quantification of immunofluorescence intensity in hundreds of ctrl and KD cells was performed for each antibody tested, and the images presented in Figure 3 are representative of this analysis. ^9^

In conclusion, we have screened sixteen VCP commercial antibodies by western blot, immunoprecipitation, and immunofluorescence by comparing the signal produced by the antibodies in human U-2 OS ctrl and *VCP* KD cells. To assist users in interpreting antibody performance, Table 4 outlines various scenarios in which antibodies may perform in all three applications. ^14^ High-quality and renewable antibodies that successfully detect VCP were identified in all applications. Researchers who wish to study VCP in a different species are encouraged to select high-quality antibodies, based on the results of this study, and investigate the predicted species reactivity of the manufacturer before extending their research.

### Limitations

Inherent limitations are associated with the antibody characterization platform used in this study. Firstly, the YCharOS project focuses on renewable (recombinant and monoclonal) antibodies and does not test all commercially available VCP antibodies. YCharOS partners provide approximately 80% of all renewable antibodies, but some top-cited polyclonal antibodies may not be available through these partners. We encourage readers to consult vendor documentation to identify the specific antigen each antibody is raised against, where such information is available.

Secondly, the YCharOS effort employs a non-biased approach that is agnostic to the protein for which antibodies have been characterized. The aim is to provide objective data on antibody performance without preconceived notions about how antibodies should perform or the molecular weight that should be observed in western blot. As the authors are not experts in VCP, only a brief overview of the protein’s function and its relevance in disease is provided. VCP experts are invited to analyze and interpret observed banding patterns in western blots and subcellular localization in immunofluorescence.

Thirdly, YCharOS experiments are not performed in replicates primarily due to the use of multiple antibodies targeting various epitopes. Once a specific antibody is identified, it validates the protein expression of the intended target in the selected cell line, confirms the lack of protein expression in the KO cell line and supports conclusions regarding the specificity of the other antibodies. All experiments are performed using master mixes, and meticulous attention is paid to sample preparation and experimental execution. In IF, the use of two different concentrations serves to evaluate antibody specificity and can aid in assessing assay reliability. In instances where antibodies yield no signal, a repeat experiment is conducted following titration. Additionally, our independent data is performed subsequently to the antibody manufacturers internal validation process, therefore making our characterization process a repeat.

Lastly, as comprehensive and standardized procedures are respected, any conclusions remain confined to the experimental conditions and cell line used for this study. The use of a single cell type for evaluating antibody performance poses as a limitation, as factors such as target protein abundance significantly impact results. Additionally, the use of cancer cell lines containing gene mutations poses a potential challenge, as these mutations may be within the epitope coding sequence or other regions of the gene responsible for the intended target. Such alterations can impact the binding affinity of antibodies. This represents an inherent limitation of any approach that employs cancer cell lines.

## Method

The standardized protocols used to carry out this KO cell line-based antibody characterization platform was established and approved by a collaborative group of academics, industry researchers and antibody manufacturers. The detailed materials and step-by-step protocols used to characterize antibodies in western blot, immunoprecipitation and immunofluorescence are openly available on Protocols.io ( protocols.io/view/a-consensus-platform-for-antibody-characterization ). ^9^ Brief descriptions of the experimental setup used to carry out this study can be found below.

### Cell lines and antibodies

The cell lines, primary and secondary antibodies used in this study are listed in Tables 1, 2, and 3, respectively. To ensure consistency with manufacturer recommendations and account for proprietary formulations (where antibody concentrations are not disclosed), antibody usage is reported as dilution ratios rather than absolute concentrations. To facilitate proper citation and unambiguous identification, all cell lines and antibodies are referenced with their corresponding Research Resource Identifiers (RRIDs). ^15, 16^ U-2 OS cells were treated with the ON-TARGETplus Human *VCP* siRNA from Horizon Discovery, cat. number L-008727-00-0005. Ctrl U-2 OS cells were treated with the ON-TARGETplus Non-targeting Control Pool, cat. number D-001810-10-05. Lipofectamine RNAiMAX (Thermo Fisher Scientific, cat. number 13778030) was used to transfect the siRNA following the manufacturer’s protocol. All cell lines used in this study were regularly tested for mycoplasma contamination and were confirmed to be mycoplasma-free.

**Table 1.** Summary of the cell lines used.

Institution	Catalog number	RRID (Cellosaurus)	Cell line	Genotype
ATCC	HTB-96	CVCL_0042	U-2 OS	WT

**Table 2.** Summary of the VCP antibodies tested.

Compagnie	Catalog number	Lot number	RRID (Antibody Registry)	Clonality	Clone ID	Host	Concentration (μg/μl)	Vendors recommended applications
Abcam	ab109198 **	GR107429-5	AB_10859334	recombinant-mono	EPR3308	rabbit	0.821	Wb
Abcam	ab109240 **	GR3176974-8	AB_10862588	recombinant-mono	EPR3307(2)	rabbit	0.350	Wb,IP,IF
Abcam	ab110308 *	GR82826-3	AB_10861815	monoclonal	3E8DC11	mouse	1.000	IP,IF
Abcam	ab36047	GR226632-1	AB_2288422	polyclonal	-	rabbit	0.300	Wb
ABclonal	A13368	81090201	AB_2760226	polyclonal	-	rabbit	1.770	Wb,IF
Aviva Systems Biology	ARP74898	QC54391-42557	AB_2936880	polyclonal	-	rabbit	0.500	Wb
Cell Signaling Technology	2648	1	AB_2214632	polyclonal	-	rabbit	0.009	Wb
Cell Signaling Technology	2649 **	2	AB_2214629	recombinant-mono	7F3	rabbit	0.034	Wb
GeneTex	GTX101089	41801	AB_1952544	polyclonal	-	rabbit	1.000	Wb,IP,IF
GeneTex	GTX113030	43187	AB_1952542	polyclonal	-	rabbit	0.300	Wb,IP,IF
GeneTex	GTX113099	40457	AB_10731852	polyclonal	-	rabbit	1.000	Wb,IF
Proteintech	10736-1-AP	83755	AB_2214635	polyclonal	-	rabbit	0.267	Wb,IP,IF
Proteintech	60316-1-lg *	10001981	AB_2881427	monoclonal	2A4B10	mouse	0.667	Wb,IF
Structural Genomics Consortium	Z-VCP-10 ** ^,A^	YSVCPA-c001	NA	recombinant-mono	Z-VCP-10	human	1.234	IP
Thermo Fisher Scientific	MA3-004 *	VG292219	AB_2214638	monoclonal	5	mouse	1.000	Wb,IP,IF
Thermo Fisher Scientific	MA5-32612 **	VL3152612	AB_2809889	recombinant-mono	JM11-15	rabbit	1.000	Wb,IF

Wb = Western blot, IP = immunoprecipitation, IF = immunofluorescence,

A = Z-VCP-10 was a gift from Susanne Gräslund (Addgene plasmid # 166559; http://n2t.net/addgene:166559; RRID:Addgene_166559).

* = monoclonal antibody,

** = recombinant antibody, NA = not available.

**Table 3.** Table of secondary antibodies used.

Company	Secondary antibody	Catalog number	RRID (Antibody Registry)	Clonality	Concentration (μg/μL)	Working concentration (μg/mL)
Thermo Fisher Scientific	HRP-Goat Anti-Rabbit Antibody (H+L)	65-6120	AB_2533967	polyclonal	1.0	0.2
Thermo Fisher Scientific	HRP-Goat Anti-Mouse Antibody (H+L)	62-6520	AB_2533947	polyclonal	1.5	0.75
Abcam	Anti-mouse IgG for IP (HRP)	ab131368	AB_2895114	monoclonal	1.0	2.0
MilliporeSigma	ANTI-FLAG ^®^ M2-Peroxidase (HRP)	A8592	AB_439702	monoclonal	1.0	0.1
Thermo Fisher Scientific	Alexa Fluor™ 555-Goat anti-Rabbit IgG (H+L)	A-21429	AB_2535850	polyclonal	2.0	0.5
Thermo Fisher Scientific	Alexa Fluor™ 555-Goat anti-Mouse IgG (H+L)	A-21424	AB_141780	polyclonal	2.0	0.5
MilliporeSigma	ANTI-FLAG ^®^ M2-Cy3™ antibody	A9594	AB_439700	monoclonal	1.0	1.0

**Table 4.** Illustrations to assess antibody performance in all western blot, immunoprecipitation and immunofluorescence.

Western blot	Immunoprecipitation	Immunofluorescence
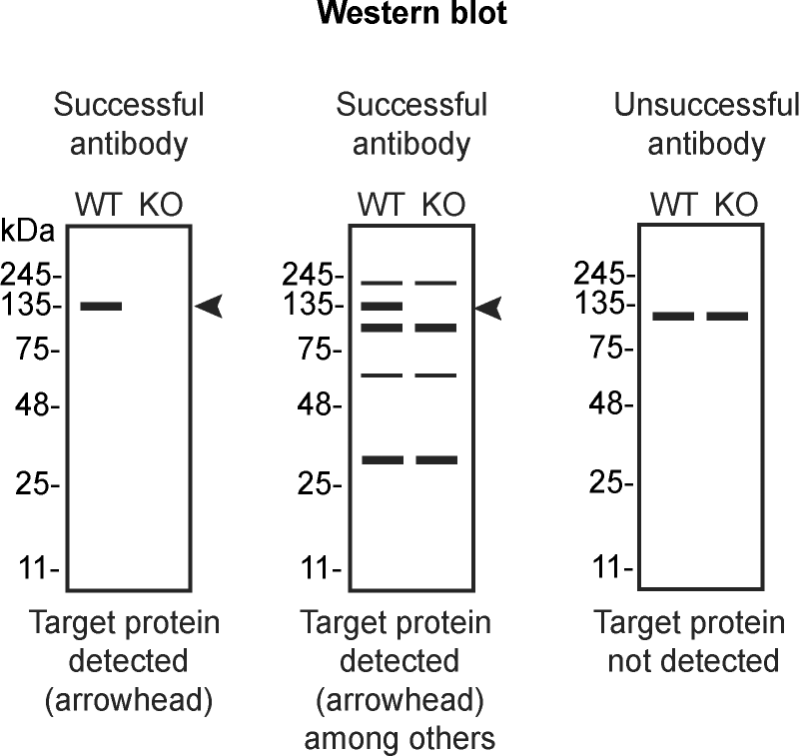	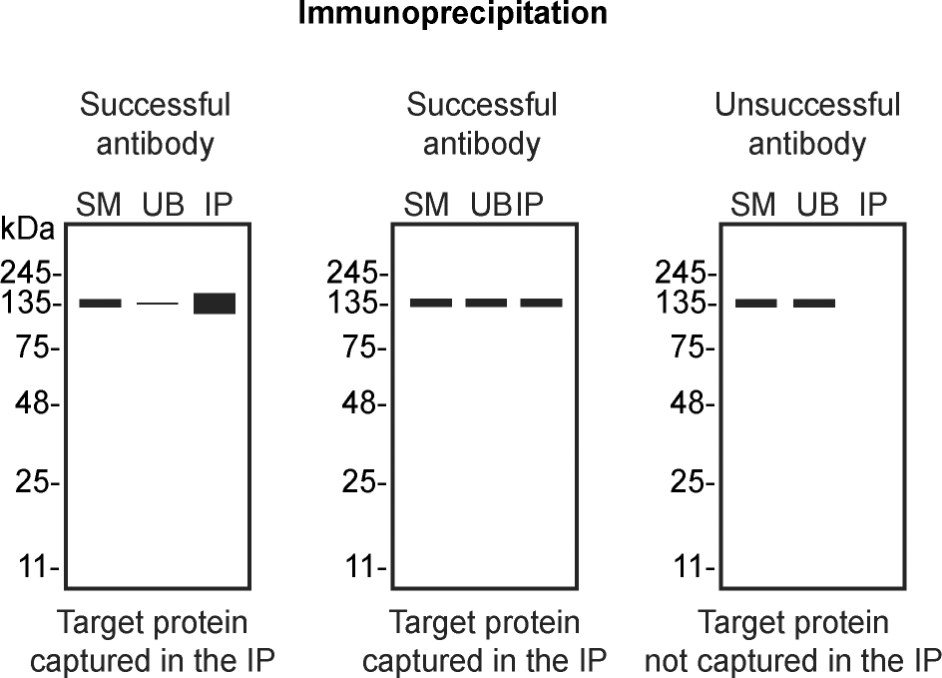	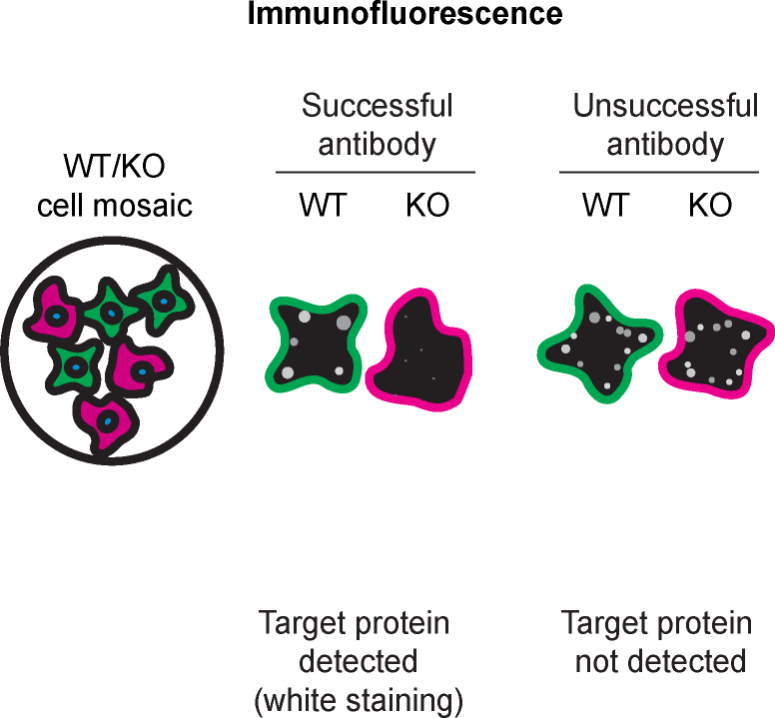

This table has been reproduced with permission from Ayoubi et al., Elife, 2023. ^14^

### Antibody screening by western blot

U-2 OS ctrl and *VCP* KD cells were collected in RIPA buffer (25mM Tris-HCl pH 7.6, 150mM NaCl, 1% NP-40, 1% sodium deoxycholate, 0.1% SDS) (Thermo Fisher Scientific, cat. number 89901) supplemented with 1x protease inhibitor cocktail mix (MilliporeSigma, cat. number P8340). Lysates were sonicated briefly and incubated 30 min on ice. Lysates were spun at ~110,000 *x g* for 15 min at 4°C and equal protein aliquots of the supernatants were analyzed by SDS-PAGE and western blot. BLUelf prestained protein ladder (GeneDireX, cat. number PM008-0500) was used.

Western blots were performed with precast midi 4-20% Tris-Glycine polyacrylamide gels (Thermo Fisher Scientific, cat. number WXP42012BOX) ran with Tris/Glycine/SDS buffer (Bio-Rad, cat. number 1610772), loaded in Laemmli loading sample buffer (Thermo Fisher Scientific, cat. number AAJ61337AD) and transferred on nitrocellulose membranes. Proteins on the blots were visualized with Ponceau S staining (Thermo Fisher Scientific, cat. number BP103-10) which is scanned to show together with individual western blot. Blots were blocked with 5% milk for 1 hr, and antibodies were incubated O/N at 4°C with milk in TBS with 0,1% Tween 20 (TBST) (Cell Signalling Technology, cat. number 9997). Following three washes with TBST, the peroxidase conjugated secondary antibody was incubated at a dilution of ~0.2 μg/ml in TBST with 5% milk for 1 hr at room temperature followed by three washes with TBST. Membranes were incubated with Pierce ECL (Thermo Fisher Scientific, cat. number 32106) prior to detection with the iBright™ CL1500 Imaging System (Thermo Fisher Scientific, cat. number A44240).

### Antibody screening by immunoprecipitation

Antibody-bead conjugates were prepared by adding 2 μg of antibody to 500 μl of Pierce IP Lysis Buffer from Thermo Fisher Scientific (cat. number 87788) in a microcentrifuge tube, together with 30 μl of Dynabeads protein A- (for rabbit antibodies) or protein G- (for mouse antibodies) (Thermo Fisher Scientific, cat. number 10002D and 10004D, respectively). 30 μl of anti-Flag M2 magnetic beads (MilliporeSigma, cat. number M8823) were used for conjugation with Z-VCP-10**. Tubes were rocked for ~1 h at 4°C followed by two washes to remove unbound antibodies.

U-2 OS WT lysates were collected in Pierce IP buffer (25 mM Tris-HCl pH 7.4, 150 mM NaCl, 1 mM EDTA, 1% NP-40 and 5% glycerol) supplemented with protease inhibitor. Lysates were rocked 30 min at 4°C and spun at 110,000 *x g* for 15 min at 4°C. 0.5 ml aliquots at 1 mg/ml of lysate were incubated with an antibody-bead conjugate for ~1 h at 4°C. The unbound fractions were collected, and beads were subsequently washed three times with 1.0 ml of IP buffer and processed for SDS-PAGE and western blot on precast midi 4-20% Tris-Glycine polyacrylamide gels.

### Antibody screening by immunofluorescence

U-2 OS ctrl and *VCP* KD cells were labelled with a green and a far-red fluorescence dye, respectively (Thermo Fisher Scientific, cat. number C2925 and C34565). The nuclei were labelled with DAPI (Thermo Fisher Scientific, cat. Number D3571) fluorescent stain. Ctrl and KD cells were plated on 96-well plate with optically clear flat-bottom (Perkin Elmer, cat. number 6055300) as a mosaic and incubated for 24 hrs in a cell culture incubator at 37 ^o^C, 5% CO _2_. Cells were fixed in 4% paraformaldehyde (PFA) (VWR, cat. number 100503-917) in phosphate buffered saline (PBS) (Wisent, cat. number 311-010-CL). Cells were permeabilized in PBS with 0,1% Triton X-100 (Thermo Fisher Scientific, cat. number BP151-500) for 10 min at room temperature and blocked with PBS with 5% BSA, 5% goat serum (Gibco, cat. number 16210-064) and 0.01% Triton X-100 for 30 min at room temperature. Cells were incubated with IF buffer (PBS, 5% BSA, 0,01% Triton X-100) containing the primary VCP antibodies overnight at 4°C. Cells were then washed 3 × 10 min with IF buffer and incubated with corresponding Alexa Fluor 555-conjugated secondary antibodies in IF buffer at a dilution of 1.0 μg/ml for 1 hr at room temperature with DAPI. Cells were washed 3 × 10 min with IF buffer and once with PBS.

Images were acquired on an ImageXpress micro confocal high-content microscopy system (Molecular Devices), using a 20x NA 0.95 water immersion objective and scientific CMOS cameras, equipped with 395, 475, 555 and 635 nm solid state LED lights (lumencor Aura III light engine) and bandpass filters to excite DAPI, Cellmask Green, Alexa-555 and Cellmask Red, respectively. Images had pixel sizes of 0.68 x 0.68 microns, and a z-interval of 4 microns. For analysis and visualization, shading correction (shade only) was carried out for all images. Then, maximum intensity projections were generated using 3 z-slices. Segmentation was carried out separately on maximum intensity projections of Cellmask channels using CellPose 1.0, and masks were used to generate outlines and for intensity quantification. ^17^ Figures were assembled with Adobe Illustrator.

## Data Availability

Zenodo: Dataset for the VCP antibody screening study, https://doi.org/10.5281/zenodo.16898393. ^18^ Data are available under the terms of the Creative Commons Attribution 4.0 International license (CC-BY 4.0).
