# Supplementary material for: A guide to selecting high-performing antibodies for VCP (UniProt ID: P55072) for use in western blot, immunoprecipitation, and immunofluorescence
Source: F1000Res. 2025 Sep 9;14:891. [Version 1] doi: 10.12688/f1000research.169502.1 (PMC12547379; doi:10.12688/f1000research.169502.1)
Supplement: Peer review history

# Reviewer report: Reviewer response for version 1

**DOI:** 10.5256/f1000research.186850.r416174

**Published:** 2025-10-22

**Authors:** Karen Bowman

**Affiliations:** 1 University of Leicester, Leicester, England, UK

The study involved characterisation of sixteen commercial antibodies, from eight different suppliers, for the Valosin-containing protein (VCP) for use in western blot, immunoprecipitation, and immunofluorescence. The study formed part of the wider YCharOS collaboration to characterise commercial antibodies for human proteins using standardised protocols with the intention of improving antibody reproducibility issues. As a Data Note, it allows the direct comparison of the performance of commercially available antibodies to VCP protein to aid scientists in choosing the most appropriate antibody for their studies. For example, in understanding the mechanisms of VCP dysfunction. This comparison would be of particular use in the fields of neuroscience and neurodegenerative diseases.

 A committee of industry and academic representatives have endorsed the platform used, and the protocols used are consistent with those in general use. The platform consisted of identification of a human cell line suitable for antibody characterisation studies i.e., with adequate levels of the protein to generate a measurable signal. They found that the commercially available U-2 OS cell line expresses VCP transcript at a level above the average range of cancer cells analysed, and therefore, was a logical choice for their study. Knockdown of the target gene using siRNA technology was used to provide appropriate negative control cells. The final step was a series of antibody characterisation procedures, limited to the most common research uses of antibodies. The interpretation of the results is left to the reader, with the study providing unbiased guidance. However, it gave some indication of the optimum dilutions of commercial antibodies to use. Limitations of the study are clearly stated.

 The summary Table 4, with illustrations to assess antibody performance in western blot, immunoprecipitation and immunofluorescence, is very helpful.

 There is a typo in sentence two. Insides should probably be insights. The calculated molecular weight of VCP, 89 kDa, is only mentioned in the legend of Figure 1, but it would be useful to have it in the text as well.

 The study is a useful starting point when choosing the most appropriate antibody for study of the VCP protein.

Are sufficient details of methods and materials provided to allow replication by others?

Yes

Is the rationale for creating the dataset(s) clearly described?

Yes

Are the datasets clearly presented in a useable and accessible format?

Yes

Are the protocols appropriate and is the work technically sound?

Yes

Reviewer Expertise:

drug discovery, biochemistry, cell biology.

I confirm that I have read this submission and believe that I have an appropriate level of expertise to confirm that it is of an acceptable scientific standard.

# Reviewer report: Reviewer response for version 1

**DOI:** 10.5256/f1000research.186850.r416175

**Published:** 2025-10-03

**Authors:** Deborah Moshinsky

**Affiliations:** 1 Institute for Protein Innovation, Boston, Massachusetts, USA

The authors tested 16 commercially available antibodies to VCP (valosin-containing protein) for their ability to detect the protein in Western Blot, Immunoprecipitation (IP), and Immunofluorescence (IF).  As negative controls, they used cells with VCP knocked down by siRNA.  Results of the Western analysis were shown using lysate from U-2 OS cells with and without siRNA knockdown (KD), and a number of antibodies indicated staining of an ~85KD protein only in cells where VCP was expressed.  Similar comparative experiments were performed for IP and IF, where lysates or cells from U-2 OS cells were compared to those from the KD cells.  At least 1 antibody of the 16 was found to recognize a protein in U-2 OS cells that was not present in KD cells, implying that the antibodies are recognizing VCP specifically.

Are sufficient details of methods and materials provided to allow replication by others?

Yes

Is the rationale for creating the dataset(s) clearly described?

Yes

Are the datasets clearly presented in a useable and accessible format?

Yes

Are the protocols appropriate and is the work technically sound?

Yes

Reviewer Expertise:

Antibody characterization and validation

I confirm that I have read this submission and believe that I have an appropriate level of expertise to confirm that it is of an acceptable scientific standard.
